# Essential Oil Composition and Anti-Cholinesterase Properties of *Cryptomeria japonica* Foliage Harvested in São Miguel Island (Azores) in Two Different Seasons

**DOI:** 10.3390/plants13233277

**Published:** 2024-11-22

**Authors:** Tânia Rodrigues, Ana Lima, Tanner Wortham, Filipe Arruda, Alexandre Janeiro, José Baptista, Elisabete Lima

**Affiliations:** 1 Institute of Agricultural and Environmental Research and Technology (IITAA), University of the Azores, 9700-042 Angra do Heroísmo, Portugal; tanyamsrod@gmail.com (T.R.); ana.pr.lima@uac.pt (A.L.); filipe.mp.arruda@uac.pt (F.A.); alex-19961917@hotmail.com (A.J.); jose.ab.baptista@uac.pt (J.B.); 2 Department of Biology (DB), Faculty of Science and Technology, University of the Azores, 9500-321 Ponta Delgada, Portugal; 3 Department of Physics, Chemistry and Engineering (DCFQE), Faculty of Science and Technology, University of the Azores, 9500-321 Ponta Delgada, Portugal; 4 The Perfumery, 621 Park East Blvd., New Albany, IN 47150, USA; twortham@theperfumery.com

**Keywords:** anti-cholinesterase activity, forest biomass valorization, brine shrimp lethality, essential oils, GC–MS analysis, seasonal variation, sustainable circular economy

## Abstract

The Azorean *Cryptomeria japonica* forest operations and wood industry generate considerable foliage biomass residues that are used for local essential oil (EO) production. However, research on seasonal variation of *C. japonica* EO remains scarce. In this study, the EOs from fresh Azorean *C. japonica* foliage (Az–CJF) collected in autumn (Aut) and spring (Spr) were obtained via hydrodistillation and investigated for their physical properties, yield, chemical composition, and bioactivities. Both EOs presented a strong odor, a yellowish color, a density around 0.9 g·mL^−1^, and similar yields (approximately 1% *v*/*w*, dry matter). Nevertheless, the GC–MS analyses showed a decrease in monoterpene hydrocarbons (MH) and an increase in oxygenated sesquiterpenes (OS) contents in Spr–EO compared with Aut–EO (16% vs. 35% for MH and 45% vs. 31% for OS, respectively). In addition, the predominant components were kaur-16-ene (23%) for Spr–EO and phyllocladene (19%) for Aut–EO, revealing that both EOs were rich in diterpene hydrocarbons (29% vs. 26%). Concerning its toxicity against brine shrimp, a low mortality (0–38%) was observed at a concentration range of 100–180 μg·mL^−1^. Regarding the anti-cholinesterase properties, both EOs were inactive against acetylcholinesterase but showed anti-butyrylcholinesterase activity superior to (–)-α-pinene, a major compound of Az–CJF EO (IC_50_ values: 84, 148, and 648 μg·mL^−1^ for Spr–EO, Aut–EO, and α-pinene, respectively). Overall, the results indicate the potential benefit of both seasonal EOs in Alzheimer’s disease treatment. In conclusion, this study demonstrated that season strongly influences the Az–CJF EO quantitative composition and thus its bioactivity, aiding in the selection of the most high-quality raw materials for use in Azorean *C. japonica* EO aromatherapy industry.

## 1. Introduction

Conifers, the dominant group of gymnosperms, are spread worldwide in diverse ecosystems, especially in the boreal and temperate forests of North America and Eurasia [1]. In fact, they have evolved intricate anatomical and chemical defense strategies against adverse abiotic conditions and against pest and pathogen attacks over a million years of co-evolution. In particular, conifers biosynthesize oleoresin (a complex terpenoid mixture of resinous and volatile compounds), typically stored in resin ducts, acting as a strong defense system [2,3,4,5,6], and possessing a great potential for commercial applications as well [1,5,6,7,8,9]. It is also noteworthy that conifer forests are major sources of biogenic volatile terpene emissions, playing an important role in atmospheric chemistry [4]. Thus, for instance, human contact with forest atmosphere (Shinrin-yoku/forest bathing) may provide a promising alternative therapy for enhancing mental health. In this context, Tsunetsugu et al. [10] reported that sensing of a weak smell of *α*-pinene, a major phytoncide in coniferous forests, induces a relaxed physiological state in humans.

Furthermore, the high timber yield generated from conifer forests, coupled with their high industrial value, constitutes a determining factor for these forests’ broad exploitation [1,6]. One of this group’s representative species is *Cryptomeria japonica* (Thunb. ex L.f.) D.Don (Japanese cedar or *sugi*), the target species of the present study. This monoecious and very large evergreen tree has a reddish-brown bark, spirally arranged leaves (needle-like) of 0.5–1 cm long, and two types of reproductive organs: female or seed cones, which retain the seeds until their maturation and ripening (by autumn period), and male or pollen cones, responsible for pollen dispersion (during spring season) [11,12].

*Cryptomeria japonica* belongs to the Cupressaceae family, which, among conifers, is one of the most economically important producers of essential oils (EOs), with interest in various industries, including complementary medicine, due to their valuable terpenoid compounds [13,14]. Currently, three main potential varieties of *C. japonica* are recognized, namely (i) var. *japonica* (*omote-sugi*), mostly found on the Pacific Ocean side of Japan; (ii) var. *radicans* (*ura-sugi*), which grows mostly on Japan’s Seaside; and (iii) var. *sinensis* (Liushan), widely distributed in several southeast China provinces (e.g., Fujian, Zhejiang, Jiangxi, and Hunan) [15]. *Cryptomeria*, as an important monotypic genus, is a challenging and stimulating plant group that deserves special attention from both taxonomists and molecular biologists, ultimately benefiting their conservation [15,16], as well as from other researchers interested in unveiling their bioactive potential for applications in the medical and biotechnology fields [17,18,19].

**Figure 1:**
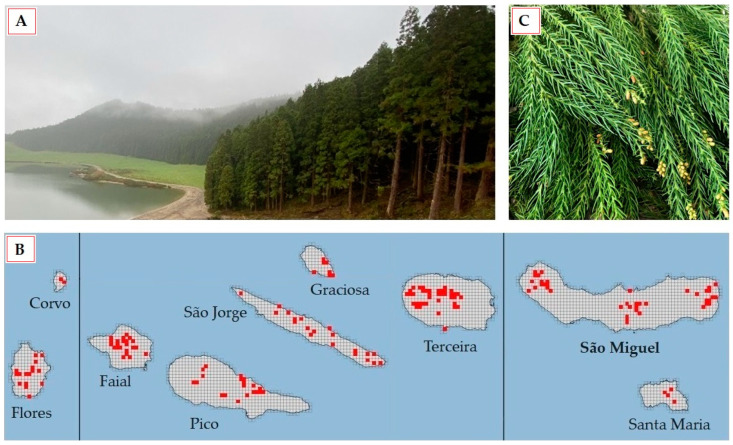
Azorean *Cryptomeria japonica*: (**A**) woodland; (**B**) current distribution (shown in red) across the three island groups of the Azores archipelago: western (Flores and Corvo), central (Faial, Pico, São Jorge, Graciosa, and Terceira), and eastern (São Miguel and Santa Maria) [22]; and (**C**) fresh foliage, the plant part utilized in this study, after removal of attached cones.

*Cryptomeria japonica* has been widely introduced into other temperate areas outside Japan and China, such as the Azores, a Portuguese archipelago composed of nine islands distributed across three groups (western, central, and eastern). Azorean forests are associated with subtropical conditions that can only be found in a few regions worldwide, which include islands with a temperate climate. In the Azores, *C. japonica*, locally known as ‘criptoméria’, was introduced from Japan in the mid-19th century and has since become the most important forestry tree, not only because its stands are a prominent component of the Azorean landscape (Figure 1A), but also due to its high economic value and role in erosion control. Currently, *C. japonica* represents over 17% of the total regional forest area and nearly 60% of the total wood-producing forest section, with major plantations being located in São Miguel Island (Figure 1B), where it occupies nearly 50% of the forest area [20,21]. As a result of a routine tree cutting cycle [20], foliage (Figure 1C) and bark are among major *C. japonica* biomass residues (CJBR) [18], which, if left unattended, can pose several environmental risks.

However, CJBR have a huge potential as sources of value-added products, as well documented over the last decades [17,18], including EOs, their fractions, and/or their individual components (EOCs) [17]. An EO, generally recognized as a safe (GRAS) product, is typically characterized as a complex liquid mixture of lipophilic and odoriferous low-molecular-weight compounds (mainly mono-, sesqui-, and diterpene hydrocarbons and their oxygenated derivatives). The EOs can be obtained by distinct extraction processes [23,24,25,26], including the traditional distillation methods, such as hydrodistillation (HD) and steam distillation (SD), frequently used at a laboratory and industrial scale, respectively [23,24]. It should be highlighted that the structural diversity of the plant terpenes/terpenoids group across the plant kingdom is mainly due to the ample catalytic diversification of plant terpene synthases (TPSs), the primary enzymes in terpenoid biosynthesis, which occurs through the mevalonate (MVA) and methylerythritol phosphate (MEP) pathways [5,8,23]. Thus, as expected, the chemical profile and yield of an EO can be highly variable, which influences its respective quality (e.g., bioactive potential) and, consequently, its specific industrial applications and price. Besides both the plant species and the extraction processes used, the major endogenous and/or exogenous factors affecting the EO chemical profile and yield of a same plant species include (i) its individual genetic variability and age; (ii) plant part and its developmental stage; (iii) surrounding abiotic and biotic environmental conditions (e.g., light, temperature, precipitation, soil nature, and microbial behavior), dependent on geographical region, climate, and seasonal variation; and (iv) management practices (e.g., daily harvest period) [3,27,28]. It should also be noted that exogenous factors, over a long period of time, might affect some of the genes responsible for volatile formation, leading to ecotypes or chemotypes in the same plant species [27]. In this context, in our recent review [17], relatively to the major EOCs of *C. japonica* foliage (CJF) EO from different geographical origins, three distinct EO chemotypes have been registered. Among these chemotypes, the α-pinene type was more frequent in Japan and European regions, such as Corsica Island and the Azores archipelago [17]. Nevertheless, although their bioactive potential may vary, all these CJF EO chemotypes have been well documented for their broad-spectrum antimicrobial [17,29] and pesticidal (e.g., anti-termite and anti-mosquito) activities [17]. Other important CJF EO bioactivities might include antioxidant [29,30]; anti-inflammatory [31]; cancer chemopreventive [32]; neuropharmacological properties (e.g., anxiolytic, analgesic [33], sedative [33,34], and relaxing [35,36] effects); and acetylcholinesterase (AChE) inhibition [37].

Currently, increasing applications and market demands for EOs [23,24,38,39,40,41] could bring unique opportunities for the sustainable management of the underutilized CJBR. Thus, studies on CJBR EO valorization remain imperative, particularly on EOs from CJF, one of the most abundant CJBR. Our previous phytochemical and bioactivity studies on EOs from Azorean CJBR (foliage, leaves, male cones, female cones, bark, and sawdust) [19,29,42,43,44] revealed that these possess interesting broad-spectrum bioactivities, including antioxidant and anti-inflammatory properties. Therefore, the evaluation of their neuroprotective potential is of great interest in the context of Alzheimer’s disease (AD), the most common form of age-related dementia worldwide, without a cure yet, and thus, the subject of major ongoing research. In fact, EOs and/or EOCs, as multi-target directed ligands (MTDLs), i.e., act on multiple targets simultaneously, coupled with their ability to cross the blood–brain barrier (BBB), may be promising candidates to combat AD [45,46]. On the other hand, some EOs can also be potentially hazardous to the public and ecosystem health [47], so evaluation of the general toxicity of CJBR EO is required. Furthermore, it is also important to highlight the scarcity of studies on the influence of seasonality over the Azorean CJBR EO chemical composition and associated biological activities.

Therefore, the present study focuses on the comparison of the chemical composition and other parameters (color, odor, density, yield, acetyl- and butyrylcholinesterase inhibition, and brine shrimp lethality) of EOs, obtained via HD from fresh Azorean *C. japonica* foliage (Az–CJF) collected from one site in São Miguel Island (Azores) in two different periods: mid-November 2022 and mid-April 2023, hereafter referred to as autumn (aut) and spring (spr), respectively. The results will aim to add more value to the *C. japonica* EO industry and, consequently, to contribute to the local circular economy through the exploitation of biomass residues generated from the typical wood processing procedures.

## 2. Materials and Methods

### 2.1. Chemicals and Reagents

A standard mixture of C7–C33 *n*-alkanes was acquired from Restek (Bellefonte, PA, USA). (–)-α-Pinene (≥97%), anhydrous sodium sulfate (Na_2_SO_4_), sodium phosphate dibasic (Na_2_HPO_4_), sodium phosphate monobasic (NaH_2_PO_4_), acetylthiocholine iodide (ATChI), butyrylthiocholine iodide (BTChI), acetone, AChE from *Electrophorus electricus L.* (electric eel), and butyrylcholinesterase (BChE) from equine serum were purchased from Sigma-Aldrich (St. Louis, MO, USA). Donepezil was obtained through Merck (Darmstadt, Germany). 5,5′-Dithiobis(2-nitrobenzoic acid) or Ellman′s reagent (DTNB) was bought from TCI (Tokyo, Japan). Dimethyl sulfoxide (DMSO) was purchased from Riedel-de Häen (Aktiengesellschaft, Seelze, Germany). Deionized water was used for all experiments.

### 2.2. Study Area and Climatic Conditions of Season Collecting Samples

The Azores is a volcanic archipelago located in the North Atlantic Ocean, about 1500 km from the Portugal mainland, stretched along 600 km in a NW–SE direction, between latitudes 36°55′43″ N and 39°43′23″ N and longitudes 24°46′15″ W and 31°16′24″ W, with a total area equivalent of 2333 km^2^ [48]. The selected island, São Miguel, belongs to the eastern Azorean group, as illustrated in Figure 1B. The oceanic temperate climate of the Azores islands is characterized by mild temperatures all year round, high relative humidity, and constant winds. During most of the year (autumn, winter, and early spring), the Azores region is frequently crossed by the North Atlantic storm track, the main path of rain-producing weather systems. On the other hand, during late spring and summer, the anticyclone influences the Azores climate, diminishing local precipitation levels [49].

**Figure 2:**
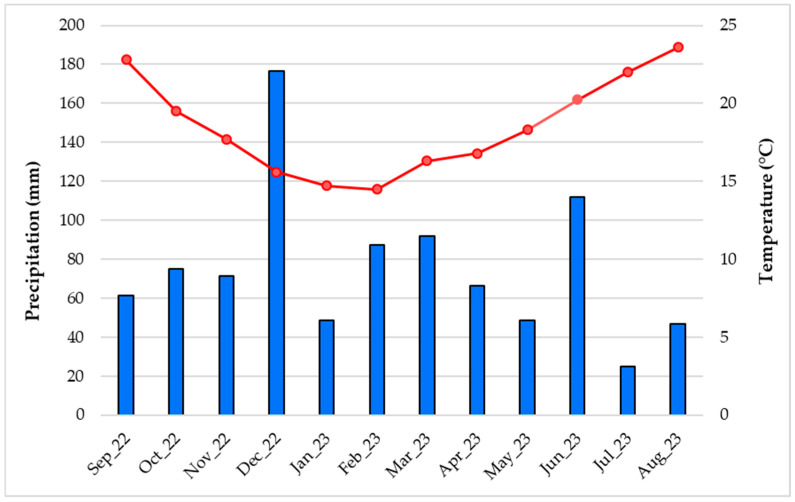
Means of the air temperature and precipitation levels by month in São Miguel Island, Azores, from September 2022 to August 2023 [50].

The average temperature and rainfall levels during the season of samples colleting ranged from 17.9 to 18.9 °C and from 79.7 to 96 mm for spring and autumn, respectively, as detailed in Figure 2. The global solar radiation (GSR) ranged from 34% to 54% and from 40% to 58% for autumn and spring, respectively. In particular, the GSR ranged from 3001.7 to 13,156.6 kJ·m^−2^ (average of 7243.6 kJ·m^−2^) and from 16,844.4 to 23,954.3 kJ·m^−2^ (average of 21,407.5 kJ·m^−2^) in November 2022 and April 2023, respectively [50]. 

### 2.3. Collection and Preparation of C. japonica Foliage Samples

The foliage of *C. japonica* was collected early morning from the same 15–20-year-old tree population located in Vila Franca do Campo (latitude 37° 44′38.347″ N, longitude 25° 21′56.263″ W, altitude 400 m), São Miguel Island (Azores archipelago, Portugal), in two seasonal periods: on 18 November 2022 (autumn; seed dispersal stage) and 13 April 2023 (spring; pollen release stage). The plant material (mature foliage from mature branchlets, or twigs, up to a maximum diameter of 10 mm) of each seasonal period was randomly taken from branch ends of ten selected *C. japonica* healthy individuals to obtain a representative foliage sample within the tree population. The collected sample was immediately brought to a laboratory at the University of the Azores and stored at –20 °C after removal of attached strobili (cones). A portion of both the fresh autumn and spring CJF samples was air-desiccated in the shade at room temperature (20 °C) until a constant weight was obtained for water content determination, revealing similar and relatively low values (55.84% and 56%, respectively).

Two voucher specimens (number AZB 4545 for autumn foliage and number AZB 4582 for spring foliage) were deposited in the Herbarium AZB–Ruy Telles Palhinha, located in the same university.

### 2.4. Extraction of Essential Oils via Hydrodistillation Process

Before HD, the CJF samples were defrosted and cut into small pieces (2–3 cm in length). The EOs were isolated using a Clevenger-type apparatus, as recommended by the European Pharmacopoeia [51]. The ratio of fresh CJF sample to water was 1:10 g·mL^−1^, and the duration of the HD was 3 h after collecting the first drop of distillate. The obtained EOs samples were dehydrated with anhydrous Na_2_SO_4_ and then stored in amber vials at 4 °C until further analysis. Each HD was performed in triplicate.

The EO content is expressed in mL per 100 g of dry matter (DM) or fresh matter (FM), and the EO density (ρ) was calculated through Equation (1), in which EO_m_ is the weight of EO and EO_v_ is the volume of EO.

(1) $$Essential\;oil\;density,\;\rho \;(g/{cm}^{3})=\frac{{EO}_{m}\;}{{EO}_{v}\;}$$

### 2.5. Gas Chromatography–Mass Spectrometry (GC–MS) Analysis of Essential Oils

The EOs were analyzed using a Shimadzu GCMS–QP2010 Ultra gas chromatograph–mass spectrometer (Shimadzu Corp., Tokyo, Japan). An aliquot of 0.1 μL of sample solution (0.1 g EO dissolved in 1 mL methylene chloride) was injected in a split mode at a 24.4:1 ratio. The carrier gas (helium) flow rate was set to 36.3 cm·s^−1^. The EOCs were separated using a ZB–5MSPlus capillary column (60 m length × 0.25 mm i.d., and film thickness of 0.25 µm) coated with 5% phenyl, 95% methyl siloxane (Phenomenex Inc., Torrance, CA, USA). The oven temperature was programmed to increase from 50 °C to 260 °C at a rate of 2 °C.min^−1^ and kept constant for 5 min at the final temperature, completing the total running time of 110 min. Both the injector and detector temperatures were 260 °C. The transfer line and ion source temperatures were 300 °C and 260 °C, respectively. The MS operated in electron ionization mode at 70 eV with a scan speed of 0.3 s and a mass range of 40–400 *m*/*z*. The identification of the EOCs was assigned as described in Arruda et al. [29] by comparing (i) their retention indices (RI), calculated according to ISO 7609 [52], relative to a homologous series of n-alkanes (C7–C33), and (ii) their recorded mass spectra with those from two MS databases: an in-house library (created with commercially available standards and components of reference EOs) and commercial libraries (FFNSC4.0, NIST11, and Wiley10). The relative percentage of each EOC was calculated by integrating the total ion current (TIC) chromatogram peaks without correction factors as the mean values of, at least, three injections from each EO sample.

### 2.6. Brine Shrimp Lethality Activity (BSLA) Assay

To assess the potential toxicity of EOs by an in vivo assay, brine shrimp lethality was used as a pre-screening method. The BSLA assay was performed according to the Solis et al. [53] method, with some modifications. Brine shrimp cysts (JBL GmbH & Co. KG, Neuhofen, Germany), purchased from a local supplier, were hatched within a period of 48 h in a container with artificial seawater at 25 °C, under constant aeration. Hatched nauplii in the second larval stage (metanauplii) were used in the bioassays. A stock solution of each EO was prepared at a concentration of 100 mg·mL^−1^ by dissolving them in DMSO. Then, this solution was diluted to 1 mg·mL^−1^ in water and sonicated. Stock solution dilutions (100, 120, 140, and 180 μg·mL^−1^) were made in 100 µL artificial seawater placed in the 96-well microplates. Control samples of artificial seawater and DMSO were also prepared to correct values with the natural mortality rate. Afterward, a 100 µL suspension of metanauplii containing 10–15 organisms was added to each well, and the covered microplate was incubated at 25 °C for 24 h. All the experiments were performed in triplicate. After 24 h, the mortality was recorded (non-motile metanauplii were considered dead) under a stereomicroscope (×12.5). Mortality data recorded were corrected according to Abbott’s [54] formula (Equation (2)):

(2) $$Mortality\;\left(\%\right)=\left[\frac{\left(L_{control}-L_{sample}\right)}{L_{control}}\right]\times 100$$

where L is the living metanauplii.

### 2.7. In Vitro Anti-Cholinesterase Assays

The AChE and BChE inhibitory activities of EOs and α-*pinene samples* were measured in vitro according to the colorimetric method developed by Ellman et al. [55], with some modifications [56]. In both assays, the tested samples were previously dissolved in DMSO and diluted in water until the desirable concentrations were achieved (concentration of DMSO in the final reaction mixtures was <0.2%). Donepezil (an approved Alzheimer medicine) was used as reference for both assays at a stock concentration of, approximately, 183 μg·mL^−1^. A control reaction was carried out using water instead of sample and considered a 100% activity standard. The absorbance (Abs) was read in a microplate reader (Thermo Scientific Multiskan FC, Waltham, MA, USA) at 405 nm. Each test was carried out in triplicate.

For anti-AChE assay, 120 µL of EO or α-*pinene* solution (1.9–960 µg·mL^−1^) and 110 µL of sodium phosphate buffer (pH 8; 0.1 M) were mixed in a microwell plate. Subsequently, 10 μL of AChE enzyme solution (0.25 U·mL^−1^) were added, and the reaction mixture was incubated for 5 min at room temperature. Finally, 10 μL of a substrate mix solution (equal parts of ATChI 75 mM and DTNB 3 mM) were added, and the resulting Abs was read after 8 min of incubation.

Regarding the anti-BChE assay, 50 µL of EO or α-*pinene* solution (1.8–909 µg·mL^−1^) and 150 µL of the aforementioned buffer were mixed in a microwell plate. Afterward, 10 μL of BChE enzyme solution (0.44 U·mL^−1^) were added, and the resulting mixture was incubated for 10 min at room temperature. Finally, 10 μL of a substrate mix solution (equal parts of BTChI 11 mM and DTNB 5 mM) were added, and the resulting Abs was read after 15 min of incubation.

The percentage inhibition of enzyme activity was calculated by comparison with the negative control using Equation (3), and the results are expressed as half-maximal inhibitory concentration (IC_50_). Lower IC_50_ value (µg·mL^−1^) means a higher anti-cholinesterase activity.

(3) $$Inhibition\;\left(\%\right)=1-\left(\frac{{Abs}_{sample}}{{Abs}_{control}}\right)\;\times 100$$

### 2.8. Statistical Analysis

Statistical analyses were performed using IBM SPSS Statistics version 28.0.1.0 software (SPSS Inc., Chicago, IL, USA). Data are represented as mean ± standard deviation (SD) of at least three independent experiments. Normal distribution of continuous variables was tested with a Shapiro–Wilk test. Paired-samples *t*-test was used for analysis of differences in the chemical and biological properties of EOs between autumn and spring seasons. Data that did not present a normal distribution were analyzed through non-parametric tests (Wilcoxon signed-rank test). Statistical significance of differences among mean values was established at *p* < 0.05.

## 3. Results and Discussion

### 3.1. Yield, Density, Color, and Odor of Essential Oils from Azorean C. japonica Foliage Harvested in Autumn and Spring

An approximate total of 900 g (divided into three distillations) of fresh Az–CJF collected from one site in São Miguel Island (Azores) in two selected seasonal periods, namely, autumn (November 2022) and spring (April 2023), which correspond to seed dispersal and pollen release plant stages, respectively, were hydrodistilled to isolate their EO.

Table 1 summarizes the results of the determination of the same parameters (color, odor, density, and yield) of the Aut–EO and Spr–EO samples. Both EOs were found to be yellow in color with a strong aromatic-fresh smell, which can be classified as a terpene-type odor [57]. In addition, both EOs are liquids with density values (around 0.9 g·mL^−1^) lower than that of water. Similarly, the yield values (*v*/*w*) of Aut–EO and Spr–EO samples (1.37% vs. 1.06% for DM, or 0.6% vs. 0.47% for FM, respectively) were not influenced by seasonal period (*t* = 2.420; *df* = 3; *p* = 0.094). However, it should be noted that, when the EOs were cooled to 4 °C for, at least, 1 h, only the Aut–EO sample developed a solid deposit with a whitish color. Concerning the yield parameter, the values reported for several EOs, obtained via HD from foliage of *C. japonica* cultivated in different regions, varied, converting them to *w/w* on a DM basis, from 0.5% to 4.7% [17,58,59,60]. Thus, the yield value of the studied EOs (1.22% and 0.96% *w/w* DM for Aut and Spr samples, respectively) is within this range. Nonetheless, it should be highlighted that comparison of data between studies is very difficult when different raw materials (e.g., growing location, plant age, and sampling time), extraction protocols (e.g., distillation time), and/or units of measurement were used, among other factors. Nevertheless, concerning the Az–CJF EOs, the yield data of a recent and systematic study of Figueiredo et al. [61] reported yield values within 0.1–0.4% (*v*/*w,* FM) for 115 EOs obtained via HD or SD from *C. japonica* aerial parts (branches and foliage with or without strobili), collected monthly for 2 years, in São Miguel, Terceira, and Pico Islands. These values were lower than those obtained in the studied seasonal periods (0.47–0.60, *v*/*w*, FM). This result may be explained, at least in part, by the different raw materials used (branches and foliage vs. foliage). In fact, previous studies reported that EO yield from CJF is higher than that of woody parts of the plant [17], such as branches. Overall, these diverse yield data indicate that Az–CJF is, typically, a good source of EO, possible to be obtained throughout the year.

It is known that climatic conditions, such as precipitation, air temperature, and solar light pattern, can strongly influence not only plant growth and development but also EO production (yield and chemical composition as well) [27]. Moreover, the effect of both temperature and light factors can accumulate because high solar radiation exposure increases leaf temperature. Furthermore, in several conifer trees, the synthesis and accumulation of terpenes appear to be highly dependent on both light intensity and temperature [4,27]. Regarding the climatic conditions of São Miguel Island, the average temperature and rainfall during the months’ colleting samples were 17.7 °C and 71.2 mm for November 2022 and 16.8 °C and 66.2 mm for April 2023, respectively (Figure 2) [50], revealing a slight decrease in temperature and precipitation in April 2023. However, in this month, the average GSR was higher when compared with November 2022 (21,407.5 and 7243.6 kJ·m^−2^, respectively) [50], showing an opposite pattern to that of temperature levels. Therefore, these data may represent, at least in part, a potential explanation for the lack of statistically significant differences in the yield values of Az–CJF EOs between the considered seasons.

In fact, Azorean forests are connected to temperate humid conditions with low thermal amplitude all year round, along with mild and relatively wet summers [20]. These authors [20] carried out a dendrochronological study in the Azores to better understand the relationships between climate and *C. japonica* growth in the archipelago, and their findings showed that precipitation variables had a relatively limited effect on the trees’ growth in comparison with temperature, which was the main and most consistent driver affecting radial growth, being this pattern similar or opposed to that of other studies (e.g., *C. japonica* from China and Japan, respectively). Thus, in a climatic change scenario where winter and summer temperatures become higher, which could lead to increased water stress, *C. japonica* may experience reduced growth rates [20]. In this context, our ongoing studies will involve the EO yield determination of CJF samples from the studied batch during different months of consecutive years, which will contribute to providing additional information for defining management approaches for *C. japonica* in the Azores.

### 3.2. Chemical Composition of Essential Oils from Azorean C. japonica Foliage Harvested in Autumn and Spring

**Figure 3:**
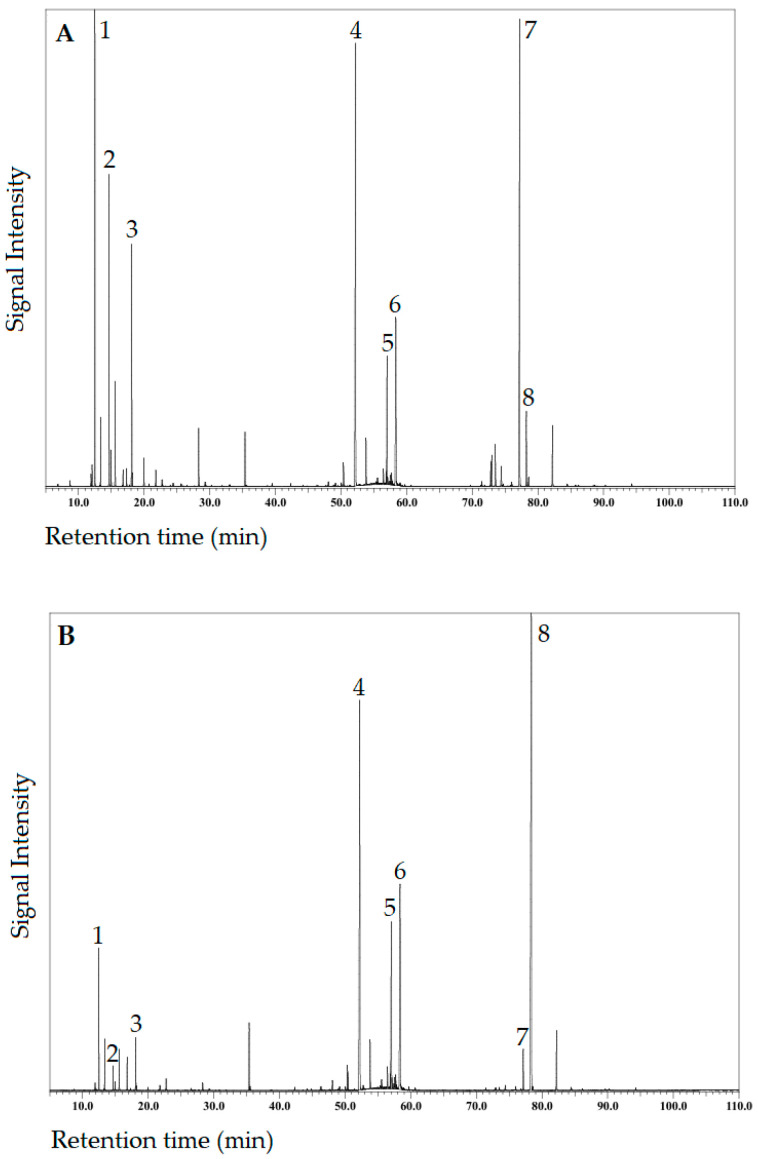
Total ion current (TIC) chromatogram on a ZB–5MSPlus capillary column of the essential oil extracted via hydrodistillation from fresh Azorean *Cryptomeria japonica* foliage collected in one site in São Miguel Island (**A**) during autumn; (**B**) during spring. Legend: 1—α-Pinene; 2—Sabinene; 3—Limonene; 4—Elemol; 5—γ-Eudesmol; 6—β + α-Eudesmol; 7—Phyllocladene; 8—Kaur-16-ene.

Figure 3A,B shows the total ion current (TIC) chromatograms, determined by GC/MS, of the Aut–EO and Spr–EO samples, respectively. Their chemical composition profile, along with the percentage of their components, are presented in Table 2, where the EOCs are listed in order of their elution from the ZB–5MSPlus capillary column and grouped into six classes: monoterpene hydrocarbons (MH), oxygenated monoterpenes (OM), sesquiterpene hydrocarbons (SH), oxygenated sesquiterpenes (OS), diterpene hydrocarbons (DH), and oxygenated diterpenes (OD).

As shown in Table 2, a total of 104 EOCs were identified in the Az–CJF EOs samples under study. Among these, 91 and 92 EOCs were present in the Aut–EO and Spr–EO samples each, accounting for 98.98% and 98.68% of the total EO composition, respectively. The results revealed that the chemical profiles of both EOs were qualitatively similar, sharing a total of 79 EOCs, whereas the relative content of some EOCs varied drastically, as discussed below.

Concerning the terpene class percentage, the Aut–EO sample was characterized by a high content of MH (35%), OS (30.8%), and DH (25.5%) and a low amount of OM (4.5%), OD (1.7%), and SH (1.6%), and, thus, its terpenes/terpenoids ratio is >1 (1.7). When compared with Aut–EO, the Spr–EO sample presented (i) the same major terpene classes, but their percentage decreased in a different order, such as OS (44.6%), DH (29.3%), and MH (15.8%); (ii) the same percentage order in the minor terpene classes, namely, OM (3.9%), OD (2.8%), and SH (2.2%); and (iii) an opposed terpenes/terpenoids ratio, i.e., <1 (0.92).

Therefore, the major EOCs (≥5%) in both EOs were MH, OS, and DH, namely phyllocladene (DH; 19%), elemol (OS; 16%), α-pinene (MH; 11.2%), sabinene (MH; 8.1%), α + β-eudesmol (OS; 7.8%), and limonene (MH; 7.4%) for Aut–EO, and kaur-16-ene (DH; 23%), elemol (20.8%), α + β-eudesmol (12.6%), α-pinene (6.5%), γ-eudesmol (OS; 6%), and phyllocladene (5.1%) for Spr–EO.

The results revealed that, with the arrival of spring, a remarkable decrease in MH content (mainly α-pinene, sabinene, and limonene) was found, namely a 2.2-fold decreased content when compared with Aut–EO. On the contrary, Spr–EO revealed an increase in OS content (mainly elemol and eudesmol isomers), namely a 1.4-fold increased content when compared with Aut–EO. In addition, a statistically significant different seasonal pattern was found in some major OM (≥2%), namely, terpinen-4-ol (3.4 times higher in Aut–EO) and bornyl acetate (1.6 times higher in Spr–EO). Biosynthetically, the monoterpenes group is derived from the MEP pathway that occurs in plant plastids, while the sesquiterpenes group is produced through the MVA pathway, located in the cytosol, although few TPSs are also found inside peroxisomes and mitochondria. However, the two terpenoid biosynthetic processes are coupled and, in general, closely associated with the photosynthetic process [64]. As reported by Malik et al. [65], the emission of volatile terpene compounds can be driven by de novo biosynthesis and/or by release from storage pools. Previous investigations on *C. japonica* from other geographical origins have demonstrated a positive correlation between the monoterpene emission rate and several abiotic environmental factors, namely light intensity [66], ozone exposure [67], and CO_2_ concentration [68]. A study by Matsunaga et al. [69] reported that *C. japonica* from Japan exhibited significant variation in monoterpene emission rates with seasonal period. Based on some of the above reported findings, we may hypothesize that the remarkable decrease in the amount of MH (highly volatile EOCs), observed in Spr–EO when compared with Aut–EO, might be associated with the highest GSR value in April, which could lead to the release of MH from CJF. However, to validate this hypothesis, the pattern emission regarding major EOCs of Az–CJF should be considered for further research. Another factor that may explain the decreased MH content in Spr–EO is related to the pollination period of *C. japonica*, occurring in the spring season, where the plant uses the greater part of the photosynthetic products, while a smaller amount is used for the biosynthesis of MH. Concerning the SH, it was found that season had no significant effect on their contents (Table 2). A previous study on the EO from Az–CJF collected during early winter in São Miguel Island [60], but in a different local than that of the present research, revealed a similar SH profile. Thus, it appears that their synthesis could mainly depend on genetic factors.

The results also revealed that the predominant component in both seasonal EOs were diterpenes, namely kaur-16-ene (23% and 2.6%) and phyllocladene (5.1% and 19%) for Spr–EO and Aut–EO, respectively, which is an unusual pattern since, typically, the major component in Az–CJF EO is the monoterpene α-pinene [e.g., 58,60,61]. In fact, the recent study of Figueiredo et al. [61], already mentioned (item 3.1.), categorized the EOs from Azorean *C. japonica* aerial parts into five distinct clusters (IA–IE), where the cluster IA, which included 79% of the total studied samples, was α-pinene type (13–43%). Among the other clusters, the group IC (8% in total samples, mainly from São Miguel Island), characterized by the presence of whitish solid deposit in the grouped EO samples, was phyllocladene type (2–22%). Therefore, this profile appears to better fit that of the studied Aut–EO sample, which also presented the mentioned solid deposit. Considering the Spr–EO sample, however, the group IE (3% in total samples, all from Terceira Island), characterized by higher levels of kaur-16-ene (21–31%), appeared to possess a more fitting profile for this EO sample. The authors [61] highlighted the larger variability in the range of some DH present in Azorean *C. japonica* EOs, a pattern already noticed for the DH in CJF from other geographical origins [70,71,72]. For instance, Yasue et al. [71] found that Japanese individual trees with kaur-16-ene or phyllocladene types are dominant in the stands of *omote-sugi* and *ura-sugi*, respectively. In another study from Appleton et al. [72], they observed that CJF from the same seed source are not uniform in their kaur-16-ene and/or phyllocladene production. Biosynthetically, cyclization of the isopimarane and pimarane tricyclic diterpene classes leads to the formation of phyllocladane (e.g., phyllocladenes) and beyerane tetracyclic diterpene classes, respectively, with rearrangements of the beyerane cation producing three classes, including kauranes (e.g., kaurenes) [72]. Interestingly, the kaurene-type diterpenes class shows a wide distribution in conifers, including Cupressaceae [73,74]. Nonetheless, from the available literature [e.g., 72], it appears that the biosynthesis of DH in this conifer family is an issue that remains unclear.

Overall, there were quantitative variations in the chemical composition of EOs from Az–CJF with season, and, as mentioned, it may be suggested that these differences are related to the plants’ growth and development, including protection from surrounding biotic and abiotic environmental stresses that can produce changes in nutrient metabolism, which can lead to variations in the plants’ secondary metabolism.

However, it is essential to recognize the limitations of this research. For instance, for each of the studied seasons, a deep knowledge of the site’s microclimatic data and soil properties is necessary, as well as of the possible interactions (positive and negative, direct and indirect) of the selected *C. japonica* batch with other surrounding organisms. In addition, to develop a more thorough knowledge of the influence of seasonal variation on the chemical composition of the Az–CJF EO under study, a broader analysis should be performed, involving the collection of CJF samples during different months of consecutive years, as already highlighted above (item 3.1).

Finally, it should be noted that the studied EOs, according to the major EOCs within the main terpene classes, i.e., DH, OS, and MH, can be classified as (i) kaur-16-ene plus phyllocladene type; (ii) elemol type; and (iii) α-pinene type, respectively, which is an unusual pattern in Az–CJF EO. Likewise, different morphological traits in foliage were observed between the two seasonal samples (data not shown). These factors sustain that further studies should be performed in order to confirm the existence of a different EO chemotype or different *C. japonica* varieties in the Azores. These studies include both needle morphological characteristics (length, curvature, and other features) and EO chemical composition analyses in individual trees from various areas of São Miguel Island, coupled with the application of molecular techniques such as Random Amplified Polymorphic DNA (RAPD) analysis.

### 3.3. Brine Shrimp Lethality Activity (BSLA) of Essential Oils from Azorean C. japonica Foliage Harvested in Autumn and Spring

Previous studies (e.g., [75]) on plant extracts reported a good correlation between toxicity results using *Artemia salina* Leach (brine shrimp) and laboratory mice. In fact, there is a growing interest in the use of brine shrimp as a model organism in preliminary assessments of a product’s toxicity to eukaryotic cells. This is due to several relevant factors, including its highly sensitivity to a variety of chemical substances. Thus, BSLA is widely used to screen a large number of extracts for drug discovery, obtained from medicinal and aromatic plants [76]. Furthermore, the great importance of this assay is related to the AChE enzyme, which has been reported as a target for *A. salina*, and, thus, the interactions between an EO and this organism may indicate a possible biological activity [77].

**Figure 4:**
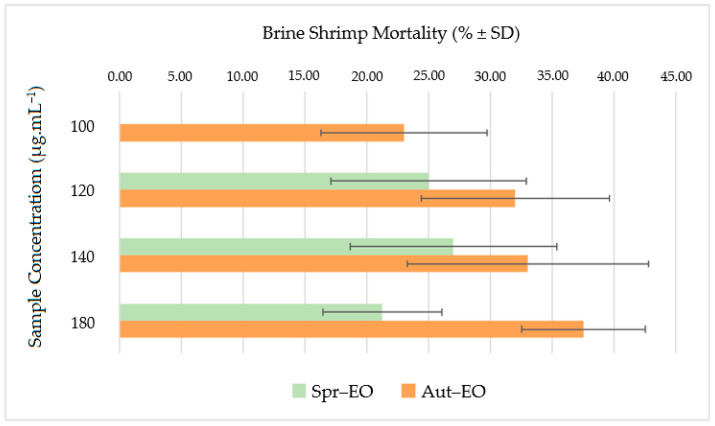
Toxicity of the essential oil (EO) extracted via hydrodistillation from fresh Azorean *Cryptomeria japonica* foliage collected in autumn (Aut) and spring (Spr) in one site in São Miguel Island. There are no statistically significant differences between samples within the used concentration range.

The potential toxicity of the studied EOs samples, estimated through a BSLA assay, is shown in Figure 4. The mortality rates varied from 0 to 27 ± 8.37% for Spr–EO and from 19 ± 7.58 to 37.5 ± 5% for Aut–EO, at a concentration range of 120–180 μg·mL^−1^.

The results indicated that both seasonal EOs did not display toxic activity (mortality rates < 50%) at the tested concentrations. However, Aut–EO was found to be more toxic, although in no statistically different manner, than the Spr–EO at all tested concentrations, particularly at 100 and 180 μg·mL^−1^ (19% vs. inactive and 37.5% vs. 21.3%, respectively). Interestingly, the limonene content of the Aut–EO sample was 3.7-fold higher than that of the Spr–EO, a factor that may, in part, explain its superior toxicity level against brine shrimp larvae. In fact, this MH has been well documented for its chemopreventive [78] and neuroprotective [79] potential.

Despite little existing research, previous studies on the BSLA of EOs from Azorean *C. japonica* samples collected in São Miguel Island, but in a different local than that of the present research, revealed interesting data. For instance, a mortality rate of 34.1% (at 100 μg·mL^−1^) was reported for the EO from CJF collected during the winter season [19], which is in accordance with that of the studied Aut–EO. Another study on the toxicity level of EOs from female cones at different developmental stages, obtained from CJF harvested during early winter, revealed low mortality rates (25.8–28.3% at 100 μg·mL^−1^) [42]. Thus, it appears that EOs from CJF, with or without female cones, can be safe for use in medical fields, such as aromatherapy.

### 3.4. In Vitro Anti-Cholinesterase Activity of Essential Oils from Azorean C. japonica Foliage Harvested in Autumn and Spring

The inhibition of AChE and BChE enzymes, which hydrolyze preferably the neurotransmitters acetylcholine (ACh) and butyrylcholine (BCh), respectively, is among the major AD therapeutic strategies. It is believed that BChE is a dominant enzyme for degradation of ACh in later stages of the disease and, thus, its inhibition is an important target to combat AD [37,45,46].

The anti-cholinesterase activity (as IC_50_ values) of the tested seasonal EO samples, as well as of the (–)-α-pinene (a major compound of Az–CJF EO), is shown in Table 3 and compared with Donepezil (a positive control sample).

Concerning the AChE inhibition, both EO samples were inactive up to the highest used concentration (0.96 mg·mL^−1^), whereas (–)-α-pinene displayed anti-AChE activity (IC_50_ = 95.7 μg·mL^−1^ or 702.5 µM), despite moderate, according to the classification of natural AChE inhibitors, based on their IC_50_ values: high potency, IC_50_ < 20 μg·mL^−1^; moderate potency, 20 < IC_50_ < 200 μg·mL^−1^; and low potency, 200 < IC_50_ < 1000 μg·mL^−1^ [80]. On the contrary, Murata et al. [37] found that Japanese CJF EO, obtained via SD, exhibited anti-AChE activity (64.8% inhibition at 100 μg·mL^−1^), but was lower compared with galanthamine hydrobromide (60.4% inhibition at 10 µM). This discrepancy in the anti-AChE activity of CJF EOs could be due to the different raw material and/or extraction protocol used, among other factors. In fact, recent research on Az–CJF EO revealed that their quantitative composition is strongly influenced by the distillation method [58].

Relatively to the inhibition of BChE, interestingly, the results indicated that both seasonal EOs showed anti-BChE activity, with Spr–EO being significantly more active than Aut–EO (IC_50_ values of 83.7 and 148.3 μg·mL^−1^, respectively). Furthermore, both EOs demonstrated a more efficient inhibition activity than (–)-α-pinene (IC_50_ = 648.4 μg·mL^−1^). To the best of our knowledge, no prior studies on the anti-BChE activity of *C. japonica* EO have been reported. Murata et al. [37] reported that nezukol and kaur-16-ene showed anti-BChE activity (IC_50_ values of 155 and >300 µM, respectively). In the present study, both nezukol and kaur-16-ene contents were higher in Spr–EO than in Aut–EO (2.7% vs. 1.6% for nezukol and 23% vs. 2.6% for kaur-16-ene, respectively), which could contribute to the superior anti-BChE activity of Spr–EO.

Although further research is needed for the identification of the bioactive EOCs of the EOs under study, as well as for determination of their potential antioxidant and anti-inflammatory properties, overall data from this research indicate that these nontoxic EOs, particularly the Spr–EO, may constitute suitable agents for AD therapy when administered repetitively through the nasal system as an aroma supplement.

## 4. Conclusions

*Cryptomeria japonica* is an EO-rich conifer widely used for industrial wood production in the Azores archipelago (Portugal). Thus, the increasing demand for EOs in the global market brings new opportunities for the valorization of major biomass residues from Azorean *C. japonica*, such as foliage, that remain underutilized, despite its EOs rich in terpenes and terpenoids with neuroprotective properties, among other important biological activities.

However, the variability of EOs terpene composition in conifers is commonly associated with intricate factors, such as spatial and temporal changes in abiotic parameters (including precipitation, temperature, and light) and biotic interactions. In particular, seasons can have a strong impact on the chemical profile of conifer’s EOs because some EOCs may be accumulated, degraded, or released at a particular period to respond to developmental and environmental stimuli, with effect on its bioactivities. In fact, the present study has shown, for the first time, that season, specifically autumn (seed dispersal stage) and spring (pollen release stage), can constitute a factor of influence over the studied Az–CJF EO bioactivities.

A high EO content (>10 mL.kg^−1^ on a DM basis) was observed in both studied EOs, namely 13.7 and 10.6 mL.kg^−1^ for Aut–EO and Spr–EO samples, respectively, revealing no significant differences between seasonal period. However, their overall chemical composition revealed some seasonal effect, mainly quantitative chemical variability. The Aut–EO demonstrated a 1.7-, 5.1-, 3.7-, and 3.4-fold increased content of α-pinene (MH), sabinene (MH), limonene (MH), and terpinen-4-ol (OM), respectively, compared with the Spr–EO. On the other hand, in Spr–EO, there is a higher accumulation of the OM bornyl acetate, along with the OS elemol and eudesmol isomers (α, β, and γ), namely, 1.6-, 1.3-, and 1.6-fold increased content when compared with Aut–EO. However, it was also observed that the studied seasonal Az–CJF EOs presented a DH as major compound (kaur-16-ene or phyllocladene for Spr–EO and Aut–EO, respectively), which is an unusual pattern since, typically, the major component in Az–CJF EO is the MH α-pinene. Thus, this chemical profile deserves further study in order to confirm the existence of different EO chemotypes or different *C. japonica* varieties in the Azores. In addition, further in-depth investigations should be performed, involving a broader collection of Az–CJF samples during different months of consecutive years.

Concerning the studied bioactivities, the results also revealed that, with the arrival of spring, the Az–CJF EO presented a slight decrease in the toxicity against brine shrimp and a significant increase in the anti-BChE activity. Nevertheless, both EOs were inactive against AChE, a factor that seems to correlate well with their weak toxic activity against brine shrimp. Thus, overall data from this research indicate that these nontoxic EOs, particularly the Spr–EO, could be considered as a potential safe neuroprotective agent for use in aromatherapy for treatment of AD-related dementia. Hence, further ongoing research will involve molecular docking studies to obtain insight into the binding affinities between the major EOCs and BChE enzyme.

On the other hand, determination of the terpene emission inventory for *C. japonica* is imperative. Therefore, further research in this area is needed to promote forest bathing therapy on the Azores archipelago and/or ecotourism.

## Figures and Tables

**Table 1:** Color, odor, density, and yield of the essential oil extracted via hydrodistillation from fresh Azorean *Cryptomeria japonica* foliage (Az–CJF) collected in autumn (Aut) and spring (Spr) in one site in São Miguel Island.

Plant Material	Essential Oil				
	Color	Odor	Density (g·mL^−1^) *	Yield (%, *v*/*w*) *	
				DM	FM
Az–CJF Aut	yellow	strong aromatic-fresh smell	0.90 ± 0.03 ^a^	1.37 ± 0.19 ^a^	0.60 ± 0.08 ^a^
Az–CJF Spr			0.91 ± 0.01 ^a^	1.06 ± 0.07 ^a^	0.47 ± 0.03 ^a^

* Results are expressed as mean ± SD (*n* = 3). Identical superscript letters in the same column indicate no statistically significant differences at *p* < 0.05. Legend: DM—dry matter; FM—fresh matter.

**Table 2:** GC–MS composition of the essential oil (EO) extracted via hydrodistillation from fresh Azorean *Cryptomeria japonica* foliage collected in autumn (Aut) and spring (Spr) in one site in São Miguel Island.

No.	Class	Component	RT	RI_L_	RI_C_	Relative Content (%)	
						Aut–EO	Spr–EO
1	MH	Tricyclene	12.03	921	918	0.25 ± 0.03	0.24 ± 0.06
2	MH	α-Thujene	12.18	924	921	0.51 ± 0.13 *	0.11 ± 0.01 *
**3**	**MH**	**α-Pinene**	12.61	932	929	11.22 ± 0.92 *	6.51 ± 0.67 *
4	MH	α-Fenchene	13.41	945	943	0.03 ± 0.01	0.03 ± 0.00
5	MH	Camphene	13.51	946	945	1.52 ± 0.18	1.58 ± 0.34
**6**	**MH**	**Sabinene**	14.80	969	967	8.12 ± 1.57 *	1.60 ± 0.15 *
7	MH	β-Pinene	15.10	974	973	0.83 ± 0.04	0.46 ± 0.03
8	MH	β-Myrcene	15.73	988	984	2.43 ± 0.29 *	1.46 ± 0.20 *
9	MH	α-Phellandrene	16.81	1002	1003	0.03 ± 0.01	0.03 ± 0.00
10	MH	δ-3-Carene	16.95	1008	1005	0.34 ± 0.03 *	1.02 ± 0.13 *
11	MH	α-Terpinene	17.47	1014	1013	0.56 ± 0.22 *	0.14 ± 0.02 *
12	MH	*p*-Cymene	17.95	1020	1020	0.05 ± 0.02	0.04 ± 0.01
**13**	**MH**	**Limonene**	18.27	1024	1025	7.43 ± 2.16 *	2.00 ± 0.30 *
14	MH	β-Phellandrene	18.36	1025	1026	0.29 ± 0.04 *	0.19 ± 0.03 *
15	MH	γ-Terpinene	20.14	1054	1053	0.90 ± 0.35 *	0.20 ± 0.07 *
16	OM	*trans*-Sabinene hydrate	20.92	1060	1065	0.05 ± 0.02	0.03 ± 0.00
17	MH	Terpinolene	21.96	1086	1080	0.47 ± 0.11 *	0.22 ± 0.06 *
18	OM	Linalool	22.90	1095	1095	0.19 ± 0.04	0.22 ± 0.09
19	OM	*cis*-Sabinene hydrate	23.00	1097	1096	0.04 ± 0.02	0.03 ± 0.02
20	OM	β-Thujone	24.17	1112	1113	0.03 ± 0.01	-
21	OM	*cis*-*p*-Menth-2-en-1-ol	24.61	1118	1119	0.09 ± 0.01 *	0.02 ± 0.00 *
22	OM	*trans*-*p*-Menth-2-en-1-ol	25.83	1136	1137	0.08 ± 0.02 *	0.02 ± 0.00 *
23	OM	Camphene hydrate	26.72	1145	1150	0.03 ± 0.01	0.06 ± 0.00
24	OM	Isoborneol	27.15	1155	1155	-	0.04 ± 0.01
25	OM	Borneol	27.88	1165	1166	0.02 ± 0.00	0.05 ± 0.00
26	OM	Terpinen-4-ol	28.50	1174	1175	2.01 ± 0.74 *	0.60 ± 0.09 *
27	OM	α-Terpineol	29.49	1186	1189	0.13 ± 0.02 *	0.09 ± 0.00 *
28	OM	*cis*-Piperitol	29.62	1195	1191	0.03 ± 0.01	-
29	OM	*trans*-Piperitol	30.45	1207	1203	0.03 ± 0.00	-
30	OM	endo-Fenchyl acetate	31.04	1218	1212	0.01 ± 0.00	0.02 ± 0.00
31	OM	Linalyl acetate	33.23	1253	1244	0.05 ± 0.00	-
32	OM	Bornyl acetate	35.58	1287	1278	1.58 ± 0.18 *	2.50 ± 0.16 *
33	OM	Isobornyl acetate	35.74	1283	1280	0.02 ± 0.00 *	0.14 ± 0.01 *
34	OM	*trans*-Sabinyl acetate	35.86	1289	1282	0.02 ± 0.00	-
35	OM	Methyl thujate	37.98	1318	1313	-	0.02 ± 0.00
36	SH	δ-Elemene	39.00	1335	1329	0.02 ± 0.00	0.02 ± 0.00
37	OM	α-Terpenyl acetate	39.72	1346	1340	0.09 ± 0.01	0.05 ± 0.00
38	SH	β-Elemene	42.54	1389	1383	0.08 ± 0.00	0.12 ± 0.01
39	SH	β-Caryophyllene	44.46	1417	1412	0.04 ± 0.01	0.05 ± 0.00
40	SH	γ-Elemene	45.08	1427	1422	0.02 ± 0.01	0.06 ± 0.00
41	SH	*trans*-β-Farnesene	46.54	1454	1446	0.03 ± 0.01	0.11 ± 0.01
42	SH	α-Humulene	46.71	1452	1448	0.02 ± 0.00	0.02 ± 0.00
43	SH	*cis*-Muurola-4(14),5-diene	47.12	1465	1455	-	0.02 ± 0.00
44	SH	10-β-H-Cadina-1(6),4-diene	47.74	1461	1465	-	0.02 ± 0.00
45	SH	*trans*-Cadina-1(6),4-diene	47.92	1475	1468	0.03 ± 0.01	0.03 ± 0.00
46	SH	Germacrene D	48.30	1484	1474	0.13 ± 0.01	0.22 ± 0.09
47	SH	β-Selinene	48.81	1489	1482	0.03 ± 0.02	0.02 ± 0.01
48	SH	*trans*-Muurola-4(14),5-diene	48.97	1493	1484	0.02 ± 0.00	0.02 ± 0.00
49	SH	α-Selinene	49.20	1498	1488	0.04 ± 0.00	0.05 ± 0.00
50	SH	α-Muurolene	49.38	1500	1491	0.10 ± 0.01	0.10 ± 0.00
51	OS	*epi*-Cubebol	49.53	1493	1493	-	0.06 ± 0.00
52	OS	β-Dihydroagarofuran	49.78	1503	1497	0.02 ± 0.00	0.02 ± 0.00
53	SH	γ-Cadinene	50.26	1513	1505	0.11 ± 0.01	0.13 ± 0.01
54	OS	Cubebol	50.38	1514	1507	0.01 ± 0.00	0.02 ± 0.00
55	SH	δ-Cadinene	50.57	1514	1510	0.93 ± 0.04	1.02 ± 0.05
56	SH	Zonarene	50.85	1526	1515	0.02 ± 0.00	0.02 ± 0.00
57	SH	*trans*-Cadina-1,4-diene	51.26	1533	1521	-	0.02 ± 0.00
58	SH	α-Cadinene	51.67	1537	1529	0.03 ± 0.01	0.04 ± 0.01
**59**	OS	**Elemol**	52.39	1548	1541	**16.00 ± 2.25** *	**20.80 ± 1.62** *
60	SH	Germacrene B	52.98	1559	1551	0.04 ± 0.00	0.11 ± 0.04
61	OS	(*E*)-Nerolidol	53.09	1561	1553	0.04 ± 0.01	0.05 ± 0.01
62	OS	Germacrene D-4-ol	54.02	1574	1568	0.97 ± 0.60	1.33 ± 0.04
63	OS	Guaiol	55.06	1597	1586	-	0.36 ± 0.00
64	OS	β-Oplopenone	55.59	1607	1595	0.09 ± 0.04	0.14 ± 0.02
65	OS	5,7-di-*epi*-α-Eudesmol	55.79	1607	1598	0.20 ± 0.06	0.26 ± 0.03
66	OS	Eudesm-5-en-11-ol	55.98	1590	1600	-	0.03 ± 0.00
67	OS	Rosifoliol	56.14	1600	1604	0.03 ± 0.03	-
68	OS	1,10-di-*Epi*-Cubenol	56.28	1618	1607	0.03 ± 0.02	0.05 ± 0.01
69	OS	10-*epi*-γ-Eudesmol	56.68	1622	1614	0.44 ± 0.04	0.70 ± 0.02
70	OS	1-*Epi*-Cubenol	56.83	1627	1616	0.03 ± 0.00	0.06 ± 0.00
71	OS	Valerianol	56.92	1623	1618	0.24 ± 0.00	-
72	OS	Agaraspirol	57.00		1619	-	0.50 ± 0.02
73	OS	Eremoligenol isomer	57.15		1622	0.22 ± 0.00	-
**74**	OS	**γ-Eudesmol**	57.23	1630	1623	3.89 ± 0.26 *	**6.04 ± 0.47** *
75	OS	Hinesol	57.67	1640	1631	0.10 ± 0.01	0.17 ± 0.01
76	OS	*τ*-Cadinol	57.79	1638	1633	0.29 ± 0.03 *	0.42 ± 0.05 *
77	OS	*epi*-α-Cadinol	57.90	1638	1635	0.34 ± 0.04 *	0.51 ± 0.06 *
78	OS	δ-Cadinol	58.04	1644	1638	0.10 ± 0.02	0.14 ± 0.04
**79/80**	OS	**β-Eudesmol + α-Eudesmol**	58.42	1649/1652	1645	**7.83 ± 0.51** *	**12.61 ± 0.81** *
81	OS	Intermedeol	58.49	1636	1646	-	0.02 ± 0.00
82	OS	Selin-11-en-4-*α*-ol	58.55	1658	1647	-	0.02 ± 0.00
83	OS	7-*epi*-α-Eudesmol	58.86	1662	1652	0.05 ± 0.01	0.06 ± 0.01
84	OS	Bulnesol	59.09	1670	1656	0.03 ± 0.00	0.07 ± 0.01
85	OS	Elemyl acetate	59.22		1659	0.09 ± 0.01	-
86	OS	Hedycaryol	59.80		1669	-	0.08 ± 0.01
87	OS	Cryptomeridiol	67.04	1813	1801	-	0.02 ± 0.00
88	OS	Oplopanonyl acetate	69.81		1855	0.02 ± 0.00	0.04 ± 0.00
89	DH	Sclarene	71.56		1891	0.03 ± 0.00	-
90	DH	Isopimara-9(11),15-diene	71.69	1905	1893	0.15 ± 0.02	0.17 ± 0.02
91	DH	Isopimara-9(11),15-diene isomer	71.88		1897	0.04 ± 0.00	-
92	DH	Rosa-5,15-diene	73.06	1926	1921	0.82 ± 0.09 *	0.03 ± 0.00 *
93	DH	Kryptomeren	73.20		1924	0.89 ± 0.12 *	0.28 ± 0.06 *
94	DH	Pimaradiene	73.75	1948	1935	1.21 ± 0.17 *	0.32 ± 0.07 *
95	DH	Sandaracopimara-8(14),15-diene	74.65	1968	1954	0.57 ± 0.09	0.30 ± 0.04
96	DH	Isophyllocladene	74.95	1966	1960	0.08 ± 0.01	0.04 ± 0.00
**97**	OD	Manool oxide	76.87	1987	1977	0.03 ± 0.00	0.03 ± 0.00
**98**	DH	**Phyllocladene**	77.43	2016	2011	**18.92 ± 2.00** *	**5.08 ± 0.62** *
99	DH	**Kaur-16-ene**	78.48	2042	2034	2.58 ± 0.22 *	**23.00 ± 2.07** *
100	DH	Abietatriene	78.83	2055	2041	0.26 ± 0.04 *	0.12 ± 0.02 *
101	OD	Nezukol	82.45	2132	2120	1.58 ± 0.52	2.70 ± 0.37
102	OD	Sandaracopimarinal	84.68	2184	2170	0.06 ± 0.04	0.08 ± 0.02
103	OD	Phyllocladanol	85.98	2209	2199	0.03 ± 0.02	0.03 ± 0.00
104	OD	*trans*-Ferruginol	90.26	2297	2299	0.02 ± 0.00	-
		Grouped components (%)					
		Monoterpene hydrocarbons (MH)				34.95 ± 6.07 *	15.83 ± 2.08 *
		Oxygenated monoterpenes (OM)				4.45 ± 1.02	3.89 ± 0.38
		Sesquiterpene hydrocarbons (SH)				1.64 ± 0.02	2.20 ± 0.23
		Oxygenated sesquiterpenes (OS)				30.76 ± 3.95 *	44.58 ± 3.24 *
		Diterpene hydrocarbons (DH)				25.49 ± 2.16	29.34 ± 2.90
		Oxygenated diterpenes (OD)				1.69 ± 0.62	2.84 ± 0.39
		Total identified components (%)				98.98 ± 0.40	98.68 ± 0.62
		Total terpenes (%)				62.08 ± 3.94 *	47.37 ± 3.88 *
		Total terpenoids (%)				36.90 ± 3.54 *	51.31 ± 2.19 *
		Ratio terpenes/terpenoids				1.68 ± 0.27 *	0.92 ± 0.10 *

Results are expressed as mean ± SD (*n* = 3). Asterisks indicate statistically significant differences (*p* < 0.05) between samples. Components higher than 5% are highlighted in boldface. Legend: RI_L_—Retention indices from the literature [62,63]; RI_C_—Retention indices calculated on a ZB–5MSPlus capillary column; RT—Retention time (minutes) values on the same column.

**Table 3:** Anti-cholinesterase activity of the essential oil (EO) extracted via hydrodistillation from fresh Azorean *Cryptomeria japonica* foliage collected in autumn (Aut) and spring (Spr) in one site in São Miguel Island.

EO and Compound	Anti-Cholinesterase Activity (IC_50,_ µg·mL^−1^)	
	Anti-Acetylcholinesterase	Anti-Butyrylcholinesterase
Aut–EO	NA	148.33 ± 44.06 c
Spr–EO	NA	83.67 ± 21.22 b
(–)-α-Pinene	95.71 ± 3.65 b	648.35 ± 22.42 d
Donepezil	0.01 ± 0.003 a	1.85 ± 0.29 a

Results are expressed as mean ± SD (*n* = 3). Different superscript letters in the same column indicate statistically significant differences at *p* < 0.05. Legend: IC_50_—half-maximal inhibitory concentration; NA—no activity.

## Data Availability

Data are contained within the article.

## Abbreviations

ACh, acetylcholine; AChE, acetylcholinesterase; AD, Alzheimer’s disease; Aut, autumn; Az–CJF, Azorean *Cryptomeria japonica* foliage; BBB, blood-brain barrier; BCh, butyrylcholine; BChE, butyrylcholinesterase; BSLA, brine shrimp lethality activity; CJBR, *Cryptomeria japonica* biomass residues; CJF, *Cryptomeria japonica* foliage; DH, diterpene hydrocarbons; DM, dry matter; EO, essential oil; EOC, essential oil component; FM, fresh matter; GC–MS, gas chromatography–mass spectrometry; GSR, global solar radiation; HD, hydrodistillation; IC_50_, half-maximal inhibitory concentration; MEP, methylerythritol phosphate; MH, monoterpene hydrocarbons; MTDLs, multi-target directed ligands; MVA, mevalonate; OD, oxygenated diterpenes; OM, oxygenated monoterpenes; OS, oxygenated sesquiterpenes; RI, retention indices; SD, steam distillation; SH, sesquiterpene hydrocarbons; Spr, spring; TIC, total ion current; TPSs, plant terpene synthases.
